# A Dictionary of Terms Used in Medicine and the Collateral Sciences

**Published:** 1845-11

**Authors:** 


*A Dictionary of Terms used in Medicine and the Collateral Sciences.* By Richard D. Hoblyn, A. M., Oxon. *First American from the second London edition. Revised with numerous additions,* by Isaac Hays, M. D., Editor of the American Journal of the Medical Sciences. 12mo. pp. 402, Philadelphia, 1845.

It is not long since, in the execution of our editorial functions, we had to animadvert in strong language on a so-called "Medical Lexicon," and to show it to be utterly unworthy of attention: it was an "original" production, strikingly so, indeed, in every respect. We have now the first American edition of a work having a similar object, which has been for years before the medical public of Great Britain; and which—under the rage for re-printing every thing that is published abroad, and thus avoiding the additional expence of copywright—appears now, for the first time, among us,—"Americanized" by the efforts of one of our own physicians. The first edition of Hoblyn's Dictionary appeared in 1835. In 1844, it passed to a second edition; and from this the edition before us has been printed.

Hoblyn's work is very different from the paltry vocabulary criticized by us some months ago. It has well founded pretensions to notice, although often sufficiently unprecise, defective, and occasionally erroneous. The whole duodecimo consists only of 402 pages; yet it professes to embrace, in addition to the "terms used in medicine," those employed also in the "collateral sciences." The searcher for information must expect, therefore, to be frequently disappointed, not only by finding omissions, but by the vagueness and insufficiency of the definitions. Still—as the respectable publishers state "its object is to serve as an introduction to the larger and more elaborate Dictionaries"—it may fulfil a useful purpose.

The first English edition contained many positive errors, most of which, we are glad to see, are corrected in the second. The American editor has added numerous terms; some of which can scarcely be regarded as appertaining exclusively to medicine and the collateral sciences, and certainly are to be found in every English Dictionary. "The Editor," he remarks, "has availed himself of many sources of information in preparing his additions, to which he need not especially refer;" but "he cannot omit to acknowledge his indebtedness to the admirable United States Dispensatory of Professors Wood and Bache, of which he has made much use, particularly in relation to the vegetable Materia Medica of the United States." Yet we think the respectable gentlemen referred to would not consent to affiliate, without proof of the parentage, some of the terms appertaining to that Materia Medica, which are to be found in the Dictionary.

In casting our eyes over the additions of the American editor, we have marked the following points, which we think worthy of notice, and correction :—

Under ACME, there is the error, doubtless typographical, of Ραραχμη, for Παραχμη. AFFERENT is said to be an "epithet given to the vessels which convey lymph to the lymphatic glands," but it is not restricted to these. We constantly speak of "*afferent* nerves," and "*afferent* blood vessels." AGRIMONIA EUPATORIA is said to be a deobstru*a*nt. ANTIRRHINUM is written *antirrhenum*. ARUM TRIPHYLLUM is written *A. triphellum*. Keratocele is placed under C instead of K. IRIDECTONICA exists for *Iridectomia*. DICTAMUS ALBUS for *Dictamnus albus*. Hooper, is given as the authority for DISPENSATORY, in the sense of "a book, which treats of the composition of medicines." It is as old as Lord Bacon. DOCIMASCIA PULMONALIS appears for *Docimasia pulmonalis*. EMPYRICS (under dogmatic) for *Empirics*. The definition of EFFERENT is liable to the same objections as apply to that of Afferent. It is said to be "a term given to vessels which convey a fluid from glands." The definition of ELABORATION is certainly not well *elaborated*. Elaboration is said to be "the different changes which assimilable [?] substances undergo by the action of the living organs, before becoming nutritive [?]." We feel that we know what this means; but assuredly not from our acquaintance with the English language. How then can the tyro comprehend the definition? GIBBOUS is said to be "an irregularity or swelling on the back or other part of the body"—instead of *Gibbosity*. Under Hardhack, SPIRÆA is printed Spirœa. Under SPIRÆA it is right. NEPETHA CATARIA, exists for *nepeta c.* In the following definition, NERVOUS and NERVOUSNESS are confounded :—" NERVOUS, belonging, or relating to the nerves; strong, vigorous; excessive irritability or mobility of the nervous system." PHILLANDRIUM is written for *Phellandrium*. PHENOMENA is said to be "any appreciable *change* which takes place in an organ or function." BALNEUM MARIS originally meant—as the American editor has it—"a saltwater bath;" but, at the present day, *Bain marie* is generally used by the French for a water bath of any kind. EXHIBEATUR means "let it be given." INC. would certainly be a faulty abbreviation for "*Incide, Cut*;" we have never met with it authoritatively; nor can JUL. for "Julepus, a julep," be considered any better. The same may be said of MAC. for "*macera, Macerate*;" and MAN. for "*manipulus*, a handful." Nor have we ever seen MI. PANIS for "*mica panis*, crumb of bread;" MIC. PANIS would be the proper abbreviation. PUBIS, "relating to the pubes," means, we apprehend, "PUBIC, relating to the pubes or pubis." PYELETIS is an evident misprint for *Pyelitis*. The definition of RATTLE is "Râle, rhoncus;" it ought to be "*rhonchus*." SABATIA ANGULARIS should be *Sabbatia angularis*; SARCOLEMMA is derived from σαρξ, 'flesh,' and λεμμα 'a coat,' not λαμμα. The latter is a typographical error in Dunglison's Dictionary. "SCABEUS, the herb of Erigeron heterophyllum and H. Philadelphicum," doubtless means "SCABIOUS, the herb of Erigeron heterophyllum and E. Philadelphicum." Under SCLEROPHTHALMIA, we are told, that "ÆTIUS applies this term to hordeolum." No such physician as Ætius ever lived. The name of the author of the Tetrabiblos is Aëtius, Αητιος. When written Ætius it is a positive error. Under SIGNATURES, DOCTRINE OF, we have "*Ars signata*" and "*cabalistic art*," as synonymes. With the former term we are not familiar. The latter cannot be a synonyme. The doctrine of signatures might be regarded as forming part of the cabalistic art; but assuredly not the whole of it. SLEEP is defined to be "the cessation of the activity of the cerebral hemispheres and ganglia of special sense, while the medulla oblongata and spinal cord is (are) in complete functional activity." This is the state of the nervous system *during sleep*, as given by Dr. Carpenter; but it is no definition of sleep. Moreover, the medulla oblongata and spinal cord are only in a state of activity in their gray portion. The medullary portion is in the same state of inactivity as "the cerebral hemispheres and ganglia of special sense." We apprehend, too, that "ganglia of special sense" required defining as much as sleep. The whole definition reminds us too much of the well known meaning of "network," given by Dr. Johnson :—"any thing reticulated or decussated, at equal distances, with interstices between the intersections."

We have made these random remarks in running over the Dictionary, which is well printed ; but doubtless some of the errors are typographical. It is difficult, indeed, to avoid them in such a work. Still, typographical errors are to be deplored in a work intended for the tyro. Of the terms used in the collateral sciences we have taken no notice. They are open, however, to the same animadversions that have been made on the others. Hoblyn's Dictionary, then, may be adapted for a limited sphere of usefulness; but it can never be a sufficient accompaniment to the student who desires to have that information on every matter appertaining to his profession, which a more complete medical dictionary affords. We certainly, therefore, cannot accord with the London Spectator—not a medical newspaper by the way—in the opinion printed on a fly leaf of the work before us, that "it is a learned, pains-taking, complete and useful work,—a Dictiontionary absolutely necessary in a medical library." It is doubtless "learned," but not "complete ;" "useful," but not "absolutely necessary ;" and as for "pains-taking"—who ever heard of a "pains-taking" dictionary !

